# Loss of Tgif Function Causes Holoprosencephaly by Disrupting the Shh Signaling Pathway

**DOI:** 10.1371/journal.pgen.1002524

**Published:** 2012-02-23

**Authors:** Kenichiro Taniguchi, Anoush E. Anderson, Ann E. Sutherland, David Wotton

**Affiliations:** 1 Department of Biochemistry and Molecular Genetics and Center for Cell Signaling, University of Virginia, Charlottesville, Virginia, United States of America; 2 Department of Cell Biology, University of Virginia, Charlottesville, Virginia, United States of America; Harvard Medical School, United States of America

## Abstract

Holoprosencephaly (HPE) is a severe human genetic disease affecting craniofacial development, with an incidence of up to 1/250 human conceptions and 1.3 per 10,000 live births. Mutations in the *Sonic Hedgehog* (*SHH*) gene result in HPE in humans and mice, and the Shh pathway is targeted by other mutations that cause HPE. However, at least 12 loci are associated with HPE in humans, suggesting that defects in other pathways contribute to this disease. Although the *TGIF1* (*TG-interacting factor*) gene maps to the *HPE4* locus, and heterozygous loss of function *TGIF1* mutations are associated with HPE, mouse models have not yet explained how loss of *Tgif1* causes HPE. Using a conditional *Tgif1* allele, we show that mouse embryos lacking both *Tgif1* and the related *Tgif2* have HPE-like phenotypes reminiscent of *Shh* null embryos. Eye and nasal field separation is defective, and forebrain patterning is disrupted in embryos lacking both Tgifs. Early anterior patterning is relatively normal, but expression of *Shh* is reduced in the forebrain, and *Gli3* expression is up-regulated throughout the neural tube. Gli3 acts primarily as an antagonist of Shh function, and the introduction of a heterozygous *Gli3* mutation into embryos lacking both *Tgif* genes partially rescues Shh signaling, nasal field separation, and HPE. Tgif1 and Tgif2 are transcriptional repressors that limit Transforming Growth Factor β/Nodal signaling, and we show that reducing Nodal signaling in embryos lacking both Tgifs reduces the severity of HPE and partially restores the output of Shh signaling. Together, these results support a model in which Tgif function limits Nodal signaling to maintain the appropriate output of the Shh pathway in the forebrain. These data show for the first time that *Tgif1* mutation in mouse contributes to HPE pathogenesis and provide evidence that this is due to disruption of the Shh pathway.

## Author Summary

Holoprosencephaly (HPE) is a devastating genetic disease affecting human brain development. HPE affects more than 1/8,000 live births and up to 1/250 conceptions. Several genetic loci are associated with HPE, and the mutated genes have been identified at some. We have analyzed the role of the *TGIF1* gene, which is present at one of these loci (the *HPE4* locus) and is mutated in a subset of human HPE patients. We show that *Tgif1* mutations in mice cause HPE when combined with a mutation in the closely related *Tgif2* gene. This provides the first evidence from model organisms that *TGIF1* is in fact the gene at the *HPE4* locus that causes HPE when mutated. The Sonic Hedgehog signaling pathway is the best understood pathway in the pathogenesis of HPE, and mutation of the *Sonic Hedgehog* gene in both humans and mice causes HPE. We show that mutations in *Tgif1* and *Tgif2* in mice cause HPE by disrupting the Sonic Hedgehog signaling pathway, further emphasizing the importance of this pathway for normal brain development. Thus we confirm *TGIF1* as an HPE gene and provide genetic evidence that *Tgif1* mutations cause HPE by disrupting the interplay of the Nodal and Sonic Hedgehog pathways.

## Introduction

Holoprosencephaly (HPE) is a prevalent human disorder affecting forebrain and craniofacial development, with an incidence of up to 1∶250 during embryogenesis, and a high frequency of intrauterine lethality [1], [2]. Recent estimates of the frequency of HPE live births are as high as 1.3 per 10,000 [3], and many children born with severe HPE phenotypes die soon after birth [4], [5]. The primary defect in HPE is a failure of ventral forebrain development with concomitant defects in midline facial structures [6], [7]. In its most severe form (alobar HPE) the forebrain fails to divide, resulting in a single brain ventricle. Less devastating forms of HPE allow near or complete separation of left and right hemispheres [8], [9]. At least 12 genetic loci have been implicated in HPE by mapping of the minimal chromosomal regions deleted in affected families [10]–[12]. Perhaps the best studied HPE gene, *Sonic hedgehog* (*SHH*), maps to the *HPE3* locus [13]. In humans heterozygous *SHH* loss of function mutations account for 17% of familial HPE and 3.7% of sporadic cases [13]–[15], suggesting a loss of function haploinsufficient phenotype [16], [17]. The genes encoding the transcription factors TGIF1, Six3 and Zic2 have been identified as the affected genes at other *HPE* loci [18]–[20]. Interestingly, recent work has shown that Six3 specifically activates expression of *Shh* in the forebrain, and in mice *Shh* and *Six3* mutations synergize to cause HPE, further emphasizing the importance of the Shh pathway [21], [22].

To establish forebrain dorsoventral patterning, the proper output of the Shh signaling pathway is essential in prechordal plate (PrCP), a primitive streak-derived axial tissue. In mouse embryos at 7.75 dpc, *Shh* expression is seen in the PrCP underlying the forebrain precursor tissue. Shh expression in the PrCP is essential for activating *Shh* expression in the overlying ventral diencephalon tissue by 9.0 dpc, where Shh specifies ventral identity [1], [23]. Gli3, a zinc-finger transcription factor that primarily acts as a repressor of Shh signaling, has been shown to play a crucial role in forebrain dorsoventral patterning. In the developing neural tissue, *Gli3* is expressed in a gradient that is higher dorsally, and *Gli3* homozygous null embryos have a forebrain with dorsally expanded ventral tissue, that lacks dorsal identity [24]–[26]. It has been shown that the proper balance between Gli3 and the ventralizing Shh is critical during forebrain patterning [25], [27]. The lack of ventral identity seen in *Shh* null embryos is partially rescued when the dose of *Gli3* is reduced genetically, suggesting that the mutual antagonism of these two factors is critical for forebrain dorso-ventral patterning. However, since the forebrain develops relatively normally in the absence of both Shh and Gli3, there must be additional pathways that specify telencephalon development, which likely depend on Foxg1 and FGF signaling [28]. Disruption of FGF signaling in the anterior by deletion of the *Fgfr1* and *Fgfr2* genes results in defective ventral telencephalon development, without disruption of the Shh signaling pathway [29].

TGIF1 (Thymine/Guanine-Interacting Factor) is a homeodomain protein, which binds directly to DNA via a thymine/guanine-containing consensus site, or interacts with Transforming Growth Factor (TGF) β-activated Smad proteins [30], [31]. In response to binding of a TGFβ family ligand to its receptors, the receptor complex phosphorylates and activates specific receptor Smad (R-Smad) proteins: Smad2 or Smad3 in the case of TGFβ, Nodal and Activin [32], [33]. Activated R-Smads complex with the co-Smad, Smad4, translocate to the nucleus and activate target gene expression via direct binding to DNA, or by interactions with other sequence specific DNA binding proteins [33]. Once recruited to DNA, a Smad complex activates transcription in part through interactions with general coactivators, such as p300/CBP [33]. The presence of specific Smad transcriptional corepressors, such as TGIF1, limits the transcriptional response by competing with coactivators and by recruiting general corepressor complexes to the Smads [31], [34]. The more recently identified TGIF2 is homologous to TGIF1 and functions similarly. TGIF2 interacts directly with DNA, or with TGFβ activated Smads and represses gene expression via the mSin3/HDAC complex, but unlike TGIF1, it does not interact with CtBP corepressors [35]–[37]. Thus overall Tgif function (TGIF1 and TGIF2) limits the magnitude of the transcriptional response to TGFβ family ligands. In addition to regulating TGFβ signaling, TGIF1 can also repress gene expression via the RXR retinoid receptor [30], [38], [39].

The *TGIF1* gene lies within the minimal *HPE4* locus, and *TGIF1* sequences were shown to be absent from individuals affected with HPE [20]. In addition to the more common deletions of *TGIF1*, single amino acid miss-sense mutations have been identified, some of which reduce transcriptional repression by TGIF1 [20], [40]–[42]. Heterozygous loss of *TGIF1* causes HPE in humans, suggesting a haploinsufficient phenotype [20]. While there is no evidence for mutations in the human *TGIF2* gene being associated with HPE, it is clearly possible that these two related proteins share overlapping functions during embryogenesis [42]. In mice, loss of *Tgif1* does not have severe phenotypic consequences, at least in a mixed strain background [38], [43]–45. In a more pure C57BL/6 strain background placental defects and reduced viability are associated with loss of Tgif1, and an intragenic mutation in *Tgif1* that may result in expression of a truncated polypeptide caused some anterior defects [46], [47]. As with *Tgif1*, *Tgif2* null mice are normal on a mixed strain background, but the combination of both mutations results in early embryonic lethality with gastrulation defects in all embryos that are homozygous null for both genes [48]. Genetically reducing Nodal signaling in these embryos reduces the severity of the gastrulation defects, consistent with an inhibitory role for Tgifs in the TGFβ/Nodal pathway. While this demonstrates an essential role for TGIF function early in embryogenesis, the function of Tgifs after gastrulation is less well understood.

Here, we investigated the role of Tgif1 and Tgif2 during forebrain development. We demonstrate that loss of Tgif function is indeed important in HPE pathogenesis, and that Tgif1 and Tgif2 play overlapping essential roles during ventral forebrain development by regulating Shh signaling. Conditional loss of function of *Tgif1* in the background of a *Tgif2* null mutation causes HPE. Furthermore, we show that the HPE phenotype is partially rescued when the dose of *Gli3* is reduced. Additionally, we show that reducing Nodal signaling reduces the severity of the HPE phenotype, and partially restores the output of the Shh pathway. This provides the first evidence that Tgifs are required for proper Shh signaling during ventral forebrain development, and verifies that *TGIF1* is a *bona fide* HPE gene.

## Results

### Loss of Tgif1 and Tgif2 causes HPE

**Figure 1:**
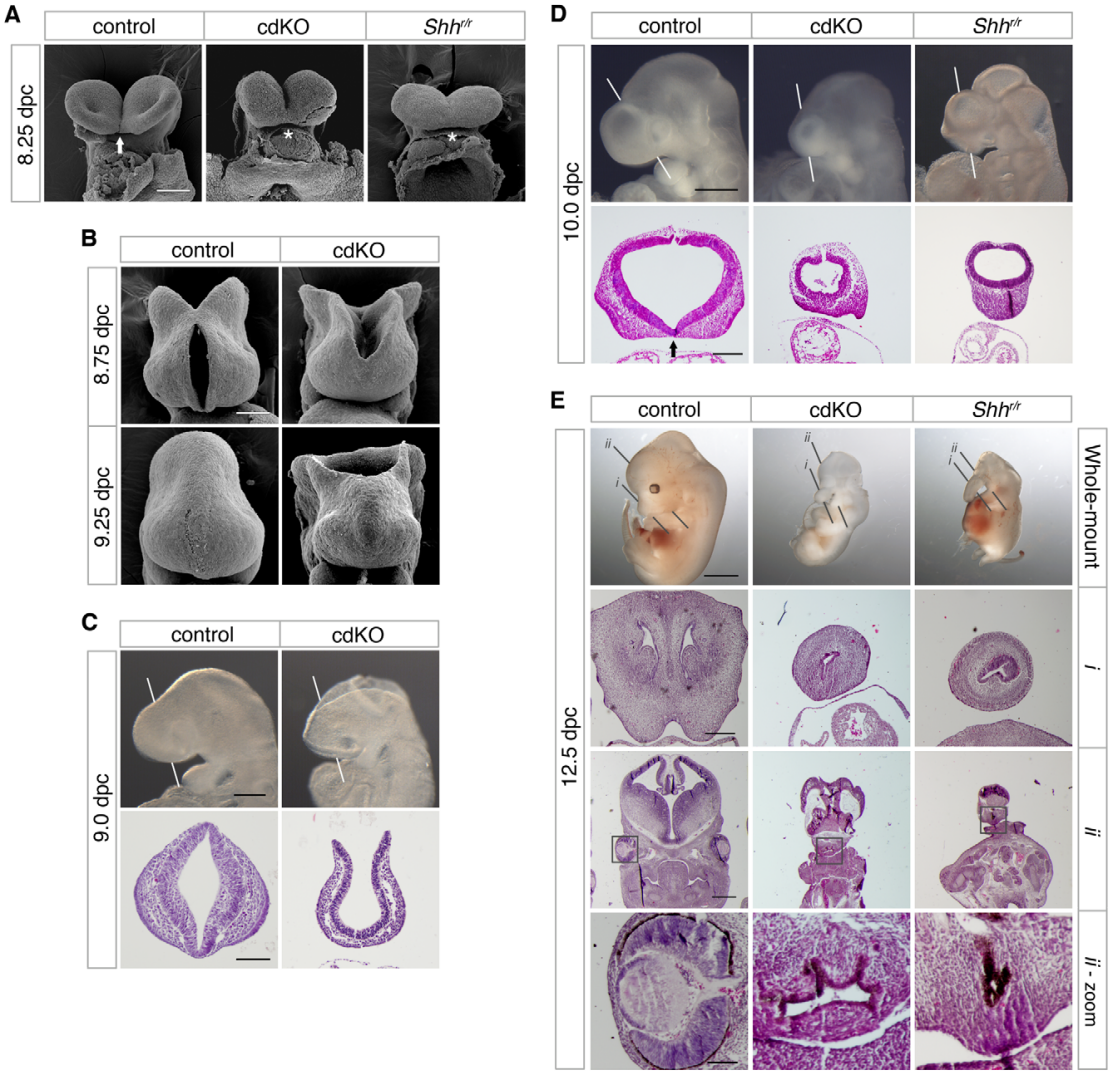
Analysis of the HPE phenotype in cdKO embryos. (A) Scanning electron microscopy (SEM) images of the frontal anterior view of embryos at 8.25 dpc, from *Tgif1;Tgif2* conditional double intercrosses with epiblast specific deletion of the conditional *Tgif1* allele (referred to as cdKO), *Shh* mutant intercrosses and a stage matched control are shown. The genotype of the control embryos is not indicated as they are representative of normal embryos from these crosses. The arrow indicates the separation of ventral lips of the cephalic folds in the control, that is defective in the cdKO and *Shh* null (marked by asterisks). Note, the conditional *Shh* null allele is referred to as ‘r’, for recombined. (B) SEM images of the frontal view of the forebrain of control and cdKO embryos at 8.75 and 9.25 dpc are shown. (C and D) Whole-mount images and hematoxylin and eosin (H&E) stained coronal section of fixed and paraffin-embedded control and cdKO embryos at 9.0 (C) and control, cdKO and *Shh^r/r^* at 10.0 dpc (D). The white lines indicate the plane of the coronal sections through the forebrain vesicle. Embryos are representative of at least 3 analyzed. In D, the division of the nasal field by the neuroepithelium is marked by an arrow. Note the continuous thickened layer of surface ectoderm in the mutants. (E) Whole mount images and H&E stained sections of fixed and paraffin-embedded control, cdKO and *Shh* null embryos at 12.5 dpc are shown. The two planes of section are indicated in the upper panels, and a magnified view of the eye is shown at the bottom (boxed region in section *ii*). Only two cdKO embryos were identified at this stage. Scale bars: 100 µm in A and B; 250 µm for whole-mount and 100 µm for section in C; 500 µm for whole-mount and 200 µm for sections in D; 2 mm for whole-mount, 250 µm for *i*, 500 µm for *ii* and 100 µm for *ii*-zoom in E.

We have previously shown that loss of both *Tgif1* and *Tgif2* results in a failure of gastrulation [48]. Conditional deletion of *Tgif1* in the epiblast, using a *loxP* flanked *Tgif1* allele [45] and the *Sox2-Cre* transgene, which is expressed in the epiblast after 5.5 dpc [49], in the background of a *Tgif2* null mutation allows these embryos (which we refer to as cdKO, for conditional double knock-out) to complete gastrulation. However, most cdKO embryos do not survive past 11.0 dpc, have left-right asymmetry defects, and have severe anterior defects. Scanning electron microscopy (SEM) analysis of frontal forebrain structure revealed that the ventral lips of the cephalic folds are fused in cdKO embryos at 8.25 dpc, as seen in *Shh* null embryos (asterisks, Figure 1A). It has been shown previously that the normally separated cephalic neural tube is fused in mouse mutants with HPE, including *Shh* null embryos [50], [51]. Additional SEM analysis at later stages shows that the midbrain neural tube fails to close in cdKO embryos even at 9.25 dpc (Figure 1B). Since human *TGIF1* mutations are associated with HPE, we next analyzed the forebrain morphology of control and cdKO embryos to determine whether there was additional morphological evidence to suggest that cdKO embryos have HPE. Whole-mount morphology of the cdKO forebrain at 9.0 dpc showed that overall forebrain size and morphology were relatively normal compared to the control. H&E staining showed that neuroepithelium and surface ectoderm were present, but that the neuroepithelium is thinner and lacks any indication of ventral morphology of the control (Figure 1C). By 10.0 dpc the cdKO forebrain was clearly abnormal, and was significantly smaller than the control (Figure 1D). Further analysis of forebrain structure by H&E staining showed that ventral forebrain morphology was defective, and that cdKO embryos appeared to have a single thickened layer of surface ectoderm in the ventral forebrain, suggesting that the nasal field has not separated by 10.0 dpc (Figure 1D). Since classic HPE phenotypes, such as cyclopia, are more apparent after 11.0 dpc, we analyzed a large number of embryos at 12.5 dpc in an attempt to identify any cdKO embryos that survive to this stage. Although the most of the cdKO embryos die by 11.0 dpc, we were able to identify two cdKO embryos that had survived to 12.5 dpc. For this analysis, we dissected a total of 117 embryos at 12.5 dpc, 76 (65%) of which appeared normal, 39 (33%) were in the process of being resorbed, and only two were doubly homozygous null for *Tgif1* and *Tgif2*. Both cdKO embryos showed cyclopia, and one of the two had developed a proboscis, similar to that in an equivalent stage *Shh* null embryo (Figure 1E). H&E staining of coronal sections through the brain tissue clearly showed that only one nasal epithelium structure was present in the proboscis tissue of both cdKO and *Shh* null embryos (Figure 1E, *i*), and that only one eye field was present in cdKO and *Shh* null embryos (Figure 1, *ii*). Thus, the morphological abnormalities in the cdKO forebrain appeared to be quite similar to those seen in *Shh* null embryos, suggesting that cdKO embryos exhibit a classic form of HPE.

### Anterior patterning in cdKO embryos

**Figure 2:**
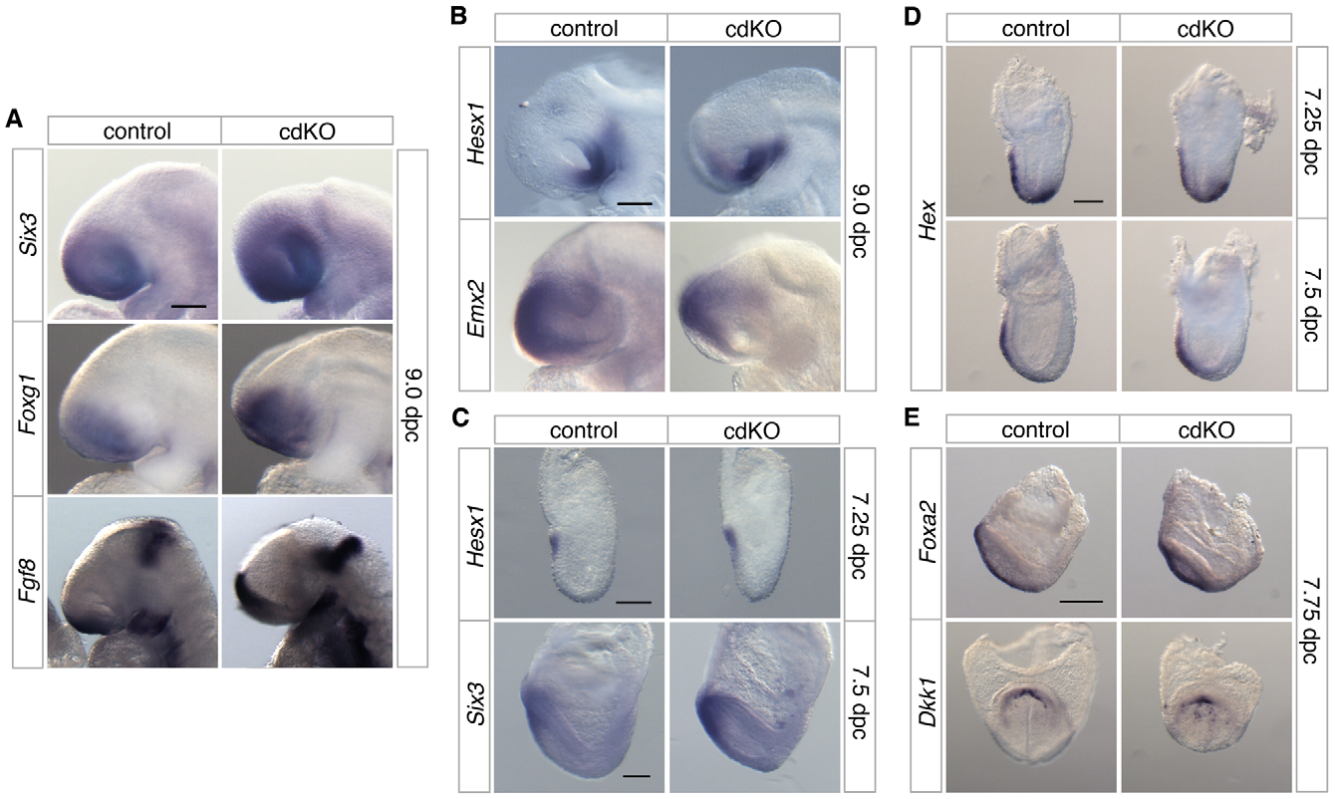
Analysis of anterior patterning in cdKO embryos. Stage matched control and cdKO embryos were analyzed by *in situ* hybridization with anti-sense probes for *Six3*, *Foxg1* and *Fgf8* at 9.0 dpc (A), *Hesx1* and *Emx2* at 9.0 dpc (B) and *Hesx1* and *Six3* at 7.25 and 7.5 dpc respectively (C). Stage matched control and cdKO embryos were analyzed at the indicated stages by *in situ* hybridization for *Hex* (D), and *Foxa2* and *Dkk1* (E). Images shown are representative of at least 3 embryos each. Scale bars: 125 µm in A, B, C and D; 250 µm in E.

The defects in forebrain structure led us to test whether anterior patterning is defective in cdKO embryos. The expression of *Six3*, a transcription factor that activates the *Shh* gene in ventral forebrain [21], [22], was seen in forebrain in both control and cdKO embryos (Figure 2A). *Foxg1*, a transcription factor that is required for proper forebrain patterning [52], is expressed in approximately the appropriate pattern in cdKO embryos (Figure 2A). Although there was no major change in the expression pattern, the expression levels of *Six3* and *Foxg1* were slightly increased in cdKO embryos. In addition, the expression of *Fgf8* was clearly increased in the cdKO forebrain, but was still present in approximately the same region as in control embryos (Figure 2A). Consistent with these observations, *Fgf8* has been shown to be a FoxH1/Smad2 target gene in the anterior, so may be up-regulated in the absence of Tgifs due to derepression of Smad dependent transcription [53]. *Hesx1*, which is a highly specific marker for ventral diencephalon [54], shows the appropriate expression pattern in the cdKO ventral diencephalon tissue at 9.0 dpc, suggesting that the midline of the ventral diencephalon is formed in cdKO embryos (Figure 2B). *Emx2*, a transcription factor that is required for dorsal forebrain patterning [55], was slightly decreased, but was present in a similar domain as in the control (Figure 2B). We next analyzed prospective forebrain tissue in younger embryos. At 7.25 dpc *Hesx1* was expressed in the anterior of both control and cdKO embryos (Figure 2C). We have shown previously that the forebrain marker, *Otx2*, was expressed in cdKO embryos at 7.5 dpc [48], and *Six3* was also expressed in the prospective forebrain tissue of cdKO embryos at early head fold (EHF) stage (Figure 2C). Taken together these results suggest that forebrain tissue is for the most part correctly patterned in cdKO embryos.

In the mouse, forebrain induction and patterning is mediated by primitive streak-derived anterior midline tissue, which includes anterior definitive endoderm (ADE) and PrCP [23], [56]. At 7.25 dpc the expression of *Hex*, a transcription factor that is essential for endoderm development [57], was seen in both control and cdKO embryos in anterior visceral endoderm and also in the ADE migrating out of the primitive streak at this stage (Figure 2D). By 7.5 dpc, *Hex* expression in anteriorly migrated ADE tissue was present, and did not appear to be significantly different between control and cdKO embryos (Figure 2D). A member of the Forkhead transcription factor family, *Foxa2*, which is normally expressed in axial tissue [58], was expressed in midline tissue of cdKO embryos at the EHF stage (Figure 2E). The PrCP can be identified by expression of *Gsc* and *Dkk1* at late head fold (LHF) stage and at 8.0 dpc [56], [59]. Appropriate expression of both *Gsc* and *Dkk1* was seen in cdKO embryos (Figure 2E and [48]), suggesting that the PrCP is present in the absence of Tgifs. This analysis suggests that anterior structures are initially patterned relatively normally in cdKO embryos.

### Shh signaling is defective in cdKO embryos

**Figure 3:**
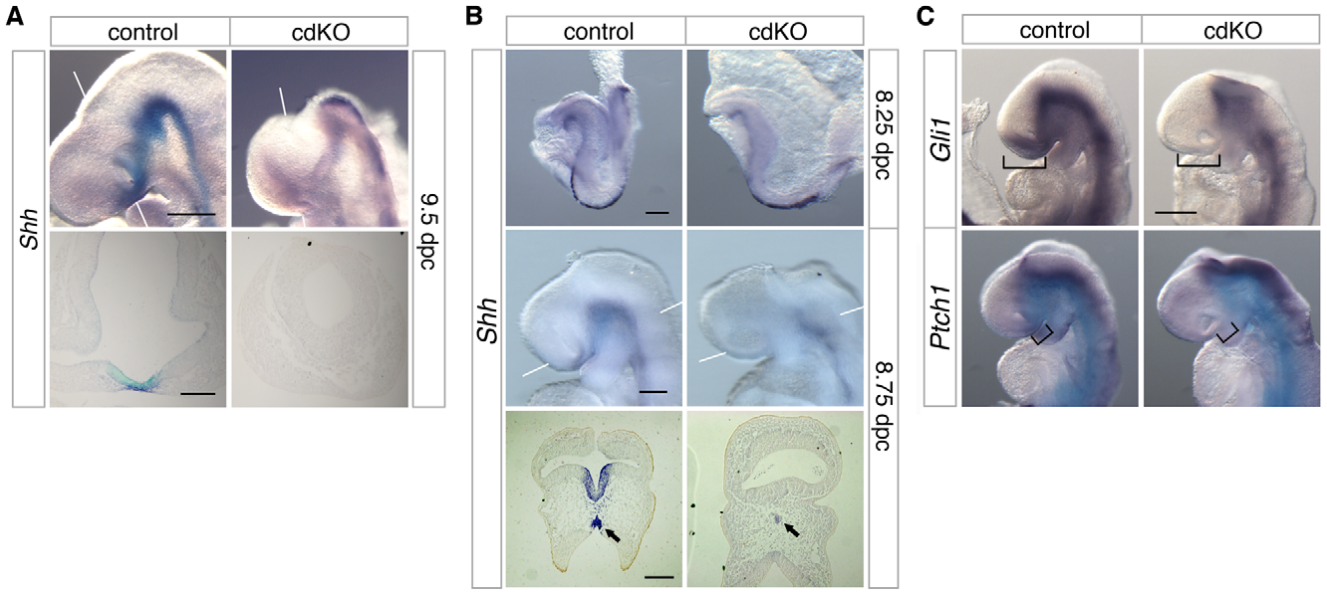
Defective Shh signaling in the forebrain of cdKO embryos. (A and B) Stage matched control and cdKO embryos at the indicated ages were analyzed by *in situ* hybridization for *Shh*. Whole mount and images of coronal sections through the forebrain vesicle of paraffin-embedded control and cdKO embryos at 9.5 dpc (A) and transverse sections through ventral forebrain and neural tube at 8.75 dpc (B) are shown. The arrows in B indicate the *Shh* expression in midline tissue. (C) Stage matched control and cdKO embryos at 9.0 dpc were analyzed by *in situ* hybridization for *Gli1* and *Ptch1*. Brackets in C indicate the expression domain that is reduced in the cdKO. White lines indicate the plane of sections. Images shown are representative of at least 3 embryos. Scale bars: 250 µm for whole-mount and 100 µm for sections in A; 125 µm for whole-mount and 100 µm for sections in B; 250 µm in C.

While there are clearly some phenotypic differences, such as the failure of the midbrain to close in cdKO embryos, the similarities between cdKO and *Shh* null embryos raised the possibility that HPE in cdKO embryos may be due to defects in the Shh signaling pathway. At 9.5 dpc, *Shh* was expressed throughout the neural tube in the floor plate, including the midline of the ventral diencephalon of control embryos (Figure 3A). However, *Shh* expression was clearly reduced in the ventral diencephalon of cdKO embryos. Similarly, in cdKO embryos *Shh* expression was reduced in the anterior midline at 8.25 dpc (Figure 3B). By 8.75 dpc *Shh* expression was present in the ventral forebrain in the control, whereas expression was clearly reduced in the cdKO ventral forebrain tissue (Figure 3B). Transverse sections showed that *Shh* expression is present but is reduced in the midline tissue including the PrCP (arrows, Figure 3B), and that *Shh* expression is not detected in the ventral forebrain (Figure 3B). We next analyzed the expression pattern of Shh signaling components at 9.0 dpc. *Ptch1* encodes a 12 transmembrane Shh receptor, and *Gli1*, a transcription factor that mediates Shh signaling [60]. Both genes are direct downstream targets of Shh signaling and are normally expressed strongly in the ventral diencephalon. In cdKO embryos the expression of *Gli1* was clearly reduced primarily in the ventral forebrain, while expression was more normal throughout the neural tube up to the forebrain-midbrain boundary (Figure 3C). *Ptch1* expression was more similar between cdKO and control embryos, although there was a slight decrease in expression in the anterior in cdKO embryos (brackets, Figure 3C). Together, these results suggest that forebrain patterning is relatively normal, but that the Shh signaling pathway is defective specifically in the ventral forebrain and PrCP. Thus it appears that Tgif function may be required for normal Shh signaling in anterior tissues.

### Shh signaling is rescued by a reduction in Gli3 levels

**Figure 4:**
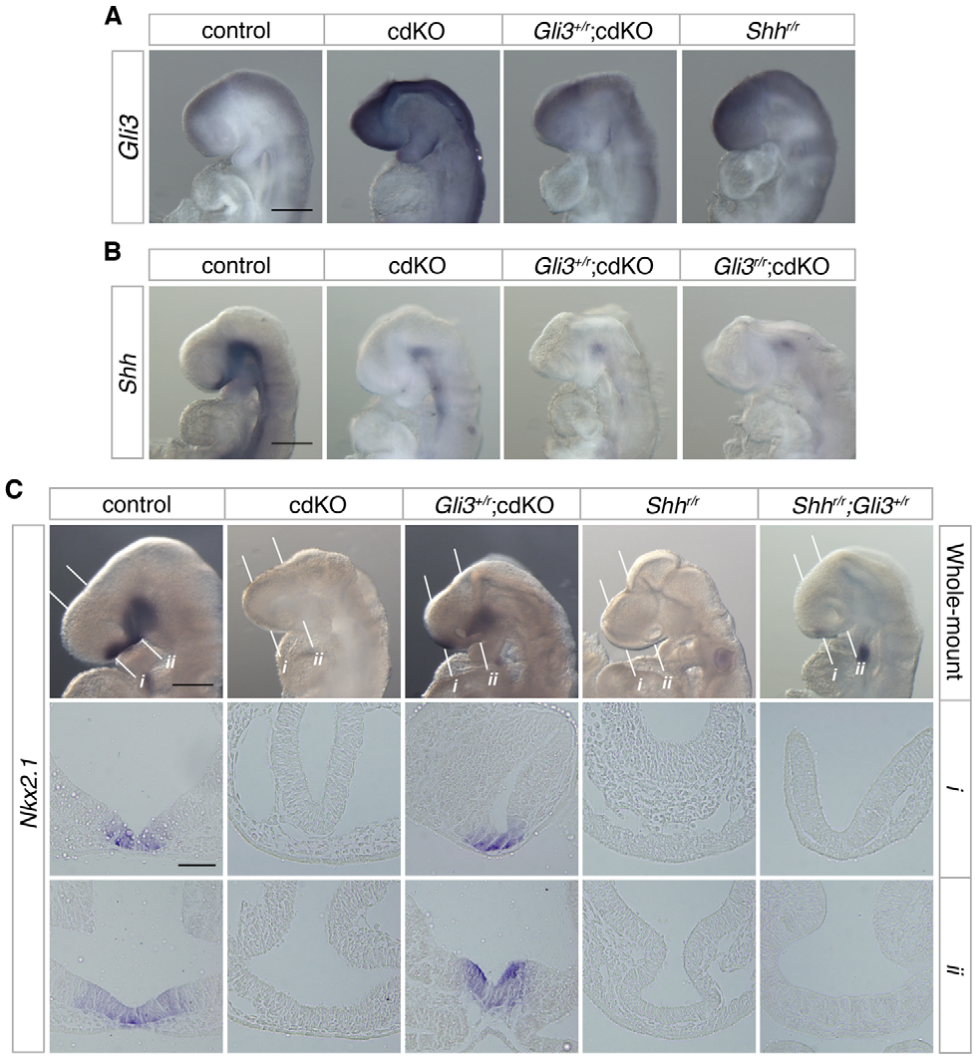
Rescue of Shh signaling by a reduction in Gli3 levels. (A) Stage matched control, cdKO, *Gli3^+/r^*;cdKO and *Shh^r/r^* embryos at 9.0 dpc were analyzed by *in situ* hybridization for *Gli3*. (B) Stage matched control, cdKO, *Gli3^+/r^*;cdKO and *Gli3^r/r^*;cdKO embryos at 9.0 dpc were analyzed by *in situ* hybridization for *Shh*. (C) Stage matched control, cdKO, *Gli3^+/r^*;cdKO, *Shh^r/r^* and *Gli3^+/r^;Shh^r/r^* embryos at 9.0 dpc were analyzed by *in situ* hybridization for *Nkx2.1*. Whole mount and coronal sections through the rostral (*i*) and caudal (*ii*) forebrain are shown. The white lines indicate the planes of the sections. Embryos shown are representative of at least 3. Scale bars: 250 µm for whole-mount and 50 µm for sections.

The transcription factor, Gli3, acts as a potent repressor of the Shh signaling pathway. In the absence of *Shh*, it has been shown that there is some increase in *Gli3* expression [24], and the HPE phenotype in *Shh* null embryos is partially rescued when *Gli3* gene dosage is reduced, suggesting that the proper balance of dorsalizing and ventralizing signals is critical during forebrain development [27], [61]. We, therefore, analyzed the expression level of *Gli3* in control and cdKO embryos. Strikingly, *Gli3* expression was clearly increased throughout the neural tube including the forebrain in cdKO embryos (Figure 4A). We also performed WISH for *Gli3* in *Shh* null embryos and compared the level of *Gli3* expression with cdKO embryos. Surprisingly, *Gli3* expression was higher in cdKO embryos than in *Shh* null embryos (Figure 4A), suggesting that there may be an additional Tgif-mediated mechanism, distinct from the reduction in *Shh* expression, that regulates *Gli3* expression.

To determine whether the increased level of Gli3 contributes to defective Shh signaling in the absence of Tgif function, we performed a genetic rescue experiment by introducing a *Gli3* mutant allele into the cdKO background. The *Gli3* allele has exon 8 flanked by *loxP* sites such that Cre-mediated recombination creates a null allele [62], which is referred to here as *Gli3^r^*. In *Gli3^+/r^*;cdKO embryos, *Gli3* expression was significantly reduced, to below the expression level seen in *Shh* null embryos (Figure 4A). In contrast, *Shh* expression was not restored in cdKO embryos that were heterozygous for *Gli3*, or in cdKO embryos that were homozygous null for the *Gli3* gene (*Gli3^r/r^*;cdKO), suggesting that the reduction in *Shh* expression is at least partially independent of *Gli3* activity in cdKO embryos (Figure 4B). We then analyzed the expression of *Nkx2.1*, a downstream target gene of Shh signaling in the forebrain 1,23, in control and a series of mutant embryos. At 9.0 dpc, the expression of *Nkx2.1* was seen in the ventral diencephalon in control embryos, whereas, *Nkx2.1* expression was not detected in cdKO or *Shh* null embryos (Figure 4C). In *Gli3^+/r^*;cdKO embryos, *Nkx2.1* expression was clearly restored while *Nkx2.1* expression in the ventral diencephalon was not rescued in *Gli3^+/r^;Shh^r/r^* embryos (Figure 4C). These results suggest that a reduction in the excess *Gli3* expression partially restores the output of the Shh signaling pathway in cdKO embryos, without affecting *Shh* expression itself.

### Reduced Gli3 levels rescue cdKO ventral forebrain morphology

**Figure 5:**
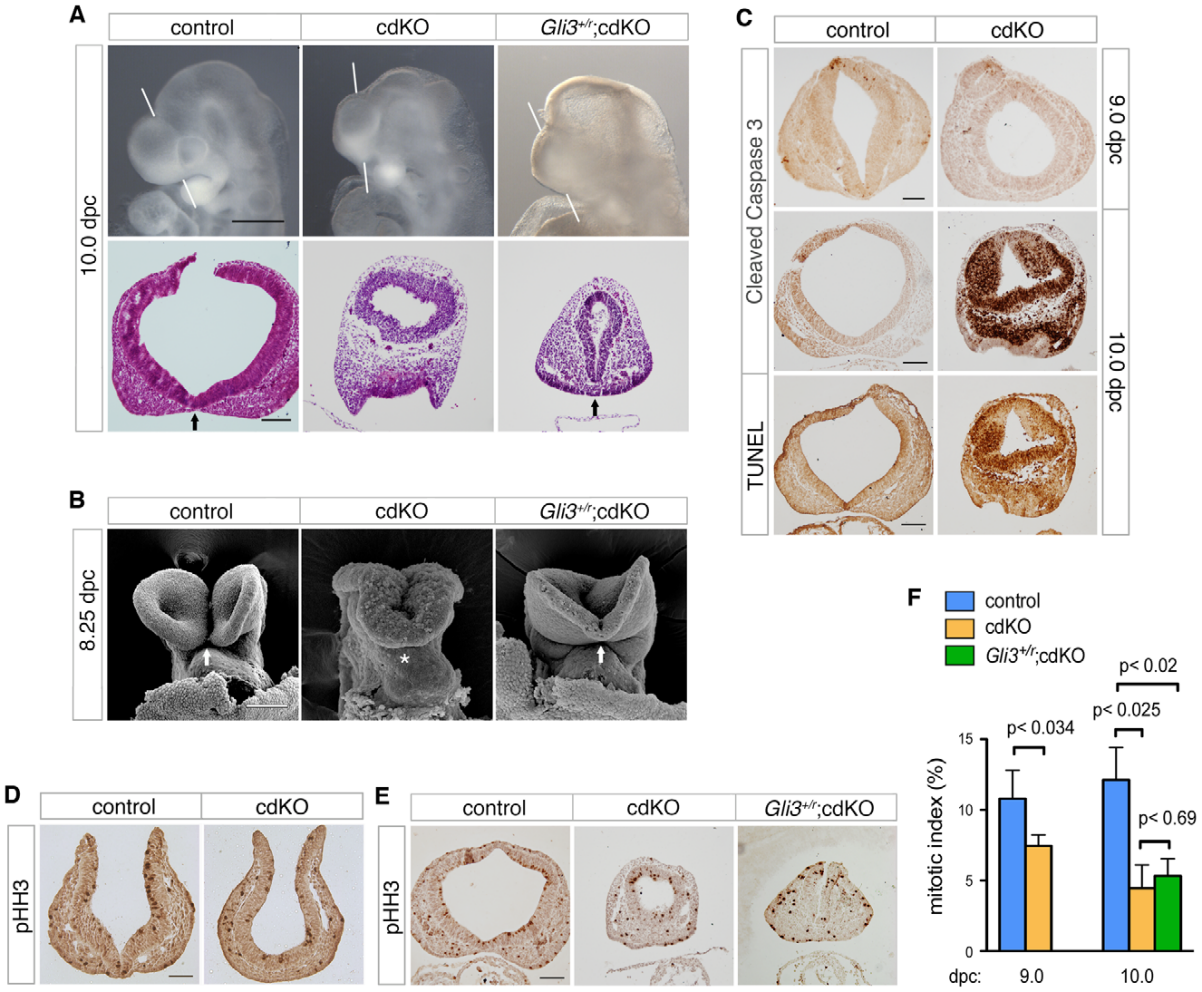
Rescued ventral forebrain structure in *Gli3* mutant cdKO embryos. (A) Whole-mount images and H&E stained coronal sections through the forebrain vesicle of control, cdKO and *Gli3^+/r^*;cdKO embryos at 10.0 dpc are shown. The white lines indicate the plane of coronal sections. Arrows indicate the division of the nasal field by the neuroepithelium. (B) SEM images of frontal anterior view of control, cdKO and *Gli3^+/r^*;cdKO are shown at 8.25 dpc. The arrows indicate the separation of the ventral lips of the cephalic folds in the control, and the partial rescue of this morphology in the *Gli3^+/r^*;cdKO, compared to the complete failure in the cdKO (asterisk). (C) Coronal sections of control and cdKO embryos at 9.0 and 10.0 dpc were analyzed by IHC with antibodies for cleaved caspase 3, or by TUNEL at 10.0 dpc. (D) Coronal sections of control and cdKO embryos at 9.0 dpc were analyzed by IHC with antibodies for Histone H3, phosphorylated on serine 10 (pHH3). (E) Coronal sections of control, cdKO and *Gli3^+/r^*;cdKO embryos were analyzed by IHC for pHH3 (F) The mitotic index of the forebrain neuroepithelium of control and cdKO embryos at 9.0 or 10.0 dpc, and of *Gli3^+/r^*;cdKO at 10.0 dpc was calculated for each section as the percentage of pHH3-stained nuclei. This data is from four control and five cdKO embryos at 9.0 dpc, and three embryos each at 10.0 dpc. Average+s.d. is shown, with the statistical significance as calculated by Student's t-test. Embryos are representative of at least 3 analyzed, unless otherwise noted. Scale bars: 50 µm for sections of 9.0 dpc embryos; 100 µm for sections from 10.0 dpc embryos.

Initial observation of *Gli3* heterozygous cdKO embryos suggests that there may be some phenotypic rescue of the cdKO phenotype. Instead of the round forebrain morphology seen in cdKO embryos, a more structured forebrain vesicle was observed in *Gli3^+/r^*;cdKO embryos at 10.0 dpc (Figure 5A). To further determine the degree of phenotypic rescue, we H&E stained coronal sections through the forebrain vesicle of control, cdKO and *Gli3* heterozygous cdKO embryos. *Gli3^+/r^*;cdKO embryos clearly had a more organized forebrain neuroepithelium morphology, and the neuroepithelium appeared to have initiated division of the nasal placode (arrows, Figure 5A), suggesting that the altered balance between *Gli3* and *Shh* expression in cdKO embryos does contribute to the HPE phenotype. In addition, SEM analysis of *Gli3* heterozygous cdKO embryos at 8.25 dpc shows a partial rescue of the forebrain structure, such that the *Gli3* heterozygous forebrain appears to be less disorganized than the cdKO, and the ventral lips of the cephalic folds appear to be partially separated in the *Gli3^+/r^*;cdKO (arrows, Figure 5B). Thus, it appears that reducing Gli3 levels results in some rescue of the cdKO phenotype. To address this further, we tested for changes in proliferation and examined forebrain patterning.

Since the anterior of the cdKO is clearly reduced in size by 10.0 dpc, we tested whether the apparent morphological rescue by *Gli3* heterozygosity might be due to a restoration of proliferation. Antibody staining for cleaved caspase 3, which is a marker of apoptotic cells, identified very few apoptotic cells in either control or cdKO forebrain at 9.0 dpc (Figure 5C). Although the cdKO embryos were still alive at 10.0 dpc, cells that were positive for cleaved caspase were present throughout the cdKO forebrain neuroepithelium, but were rarely seen in the control (Figure 5C). Consistent with this, TUNEL analysis showed increased apoptosis in the cdKO forebrain at 10.0 dpc (Figure 5C). To determine whether proliferation is reduced in cdKO embryos, we stained multiple coronal sections of control and cdKO forebrains at 9.0 and 10.0 dpc with an antibody to Histone H3, phosphorylated on serine 10 (pHH3), which is a marker for cells in late G2 and mitosis. Mitotic cells were seen throughout neuroepithelium for both control and cdKO at 9.0 dpc (Figure 5D). Quantification of the proportion of mitotic cells in the neuroepithelium showed that there was a significant reduction in proliferation at 9.0 dpc, that was more pronounced by 10.0 dpc (Figure 5E and 5F). These results suggest that cdKO embryos have proliferation defects in the forebrain neuroepithelium, and that the reduced proliferation is seen prior to any increase in apoptosis. We next tested whether the apparent rescue of forebrain morphology in *Gli3^+/r^*;cdKO embryos was accompanied by a restoration of normal levels of proliferation. However, in *Gli3^+/r^*;cdKO embryos, proliferation levels were not different from the cdKO at 10.0 dpc (Figure 5E and 5F). This suggests that the phenotypic rescue in *Gli3^+/r^*;cdKO embryos is independent of changes in proliferation, and that the morphological defects in the cdKO are not solely due to reduced proliferation.

**Figure 6:**
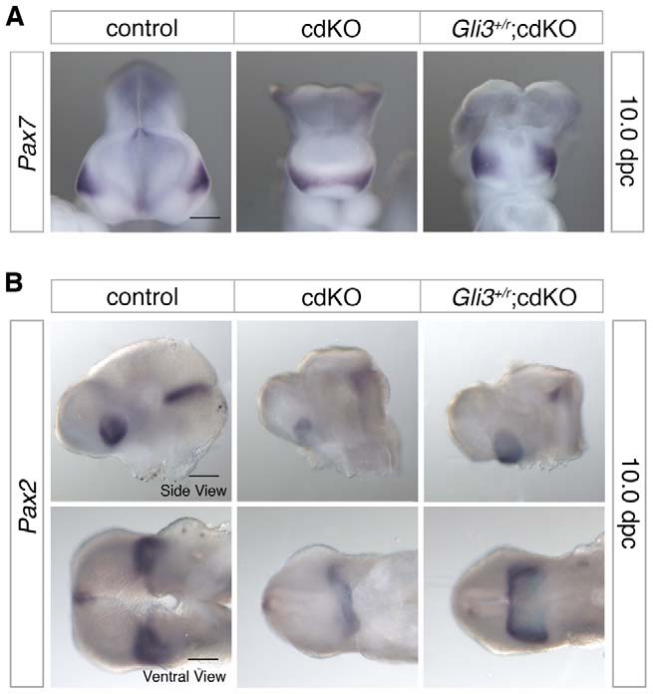
Defective separation of facial features. (A) Frontal forebrain images of stage matched control, cdKO and *Gli3^+/r^*;cdKO embryos analyzed by *in situ* hybridization for *Pax7*. (B) Side and ventral views of embryos analyzed for *Pax2* expression are shown. The *Gli3^+/r^*;cdKO embryos shown in A and B are representative of 7 and 4 embryos respectively, other images are representative of at least 3. Scale bars: 250 µm for *Pax2* and *Pax7* side view, and 200 µm for *Pax2* ventral view.

To further characterize ventral structure, we analyzed the expression pattern of *Pax7*, a nasal field marker, as well as the eye field marker, *Pax2* [61]. Normally by 10.0 dpc, the nasal field is well separated as evidenced by the position of the ventral neuroepithelium clearly separating the facial field (see Figure 1D, for example). In *Shh* null embryos, *Pax7* expression is present in a single central region suggesting that the nasal field is not fully separated, whereas when the dose of *Gli3* is reduced in *Shh* null embryos *Pax7* expression becomes separated to the two nasal fields [61]. In cdKO embryos, *Pax7* expression was observed as a single continuous band, suggesting that nasal field separation is defective (Figure 6A). In *Gli3^+/r^*;cdKO embryos, *Pax7* expression was clearly well separated and was more similar to that seen in controls, suggesting that the nasal field separation defect is partially rescued in *Gli3* heterozygous cdKO embryos (Figure 6A). Similarly, *Pax2* expression was reduced and was seen as a single continuous band in cdKO embryos, suggesting that eye field separation is defective (Figure 6B). In *Gli3^+/r^*;cdKO embryos, the *Pax2* expression level was increased, and appeared as less of a continuous band with distinct eye fields on both sides of the forebrain (Figure 6B). These results suggest that the increase in *Gli3* expression, and the altered balance between Gli3 and Shh contribute to the HPE phenotype seen in cdKO embryos resulting in a disruption of the separation of facial primordia.

### Nodal dependence of forebrain development in the cdKO embryos

The TGFβ/Nodal signaling pathway has been linked to HPE pathogenesis. For example, HPE has been reported in mouse mutants that result in reduced TGFβ/Nodal signaling, such as *Nodal;Smad2* double heterozygotes [63]. Since mutations in these genes result in a reduction in the output of TGFβ/Nodal signaling, rather than the expected increase in cdKO embryos, we generated mice that are heterozygous for both *Nodal* and *Smad2* genes for comparison to our cdKOs. The *Smad2* null allele is referred to here as ‘r’ and the *Nodal* null allele as ‘z’ (see Materials and Methods for a full explanation). Of 41 *Nodal;Smad2* double heterozygotes analyzed between 10.5 and 12.5 dpc only one had HPE, although an additional 15 of the 41 double heterozygotes had anterior truncations or a severe growth delay. The *Nodal;Smad2* double heterozygous embryo with HPE had a proboscis and a partial failure to separate the eyes, but was significantly larger than cdKO and *Shh* null embryos (Figure S1). H&E staining of sections through the nasal structure showed a single nasal epithelium that appears structurally similar to that of cdKO and *Shh* null embryos (Figure S1, *i*). H&E staining of sections through the eye field showed that a laterally elongated, large optic structure containing two distinct eyes had begun to form, while cdKO and *Shh* null embryos had only one small pigmented eye field vesicle (Figure S1, *ii*). Thus, in contrast to the cdKO embryos, it appears that in embryos with reduced Nodal pathway activity HPE is relatively rare.

**Figure 7:**
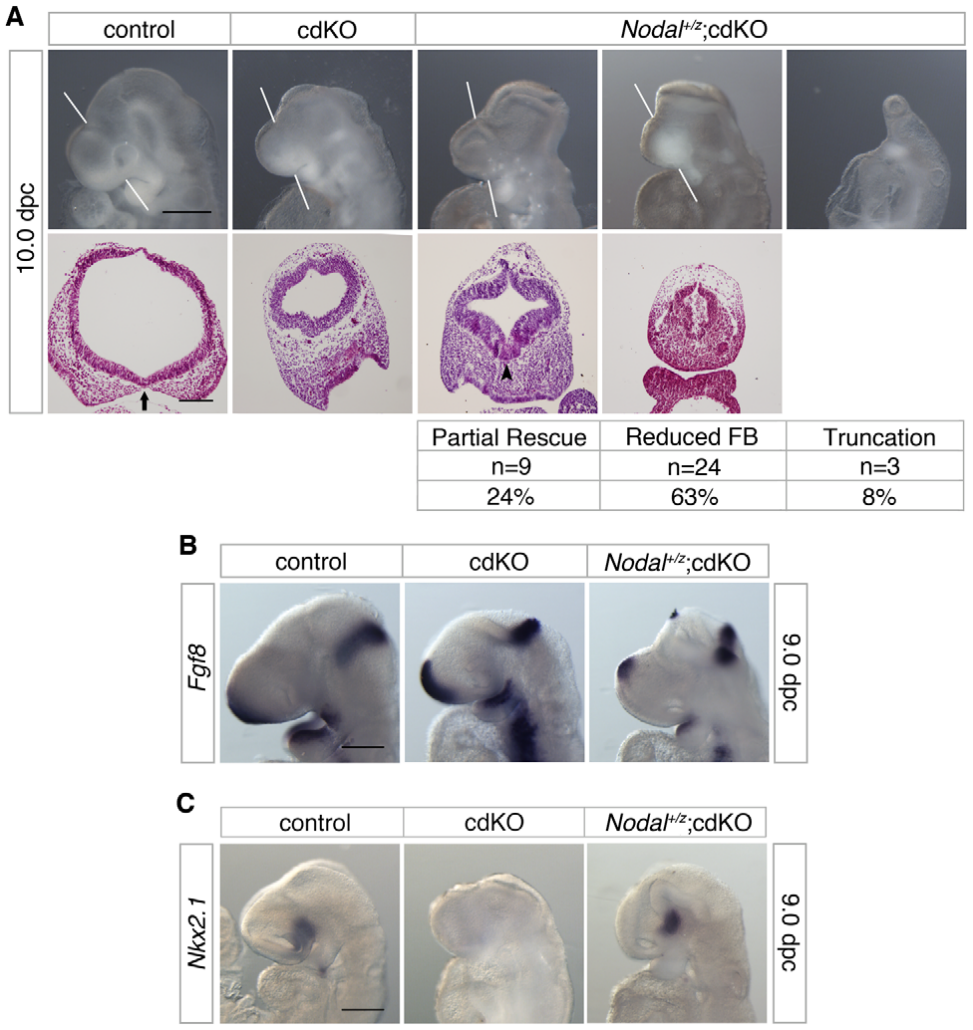
Effects of Nodal heterozygosity of the cdKO phenotype. (A) Whole-mount images and H&E stained coronal sections through the forebrain vesicle of control, cdKO and *Nodal^+/z^*;cdKO embryos at 10.0 dpc are shown. The white lines indicate the plane of coronal sections. Three *Nodal^+/z^*;cdKO embryos are shown that are representative of the three classes of phenotype seen. The numbers of *Nodal^+/z^*;cdKO embryos analyzed at 10.0 dpc (from a total of 317 embryos) are shown below for each class of phenotype, together with the percentage of the *Nodal^+/z^*;cdKO embryos with each phenotype: Partial rescue of the HPE phenotype; Reduced forebrain (FB); and severe truncation. Two additional embryos were too severely delayed to be classified. Note the improved ventral neuroepithelium morphogenesis in the left hand *Nodal^+/z^*;cdKO embryo (arrowhead). The separation of the facial field by the neuroepithelium in the control is indicated by an arrow. (B) Control, cdKO and *Nodal^+/z^*;cdKO embryos at 9.0 dpc were analyzed for *Fgf8* expression, and for *Nkx2.1* expression in (C). Embryos in B and C are representative of at least three each. Scale bars: 250 µm for whole-mount and 100 µm for sections in A; 250 µm in B and C.

Our previous analysis of *Tgif1;Tgif2* double null mutants showed that Tgifs limit Nodal signaling [48]. To test whether the HPE phenotypes in cdKO embryos were due to increased Nodal signaling, we generated cdKO embryos that carry a *Nodal* heterozygous mutation. Initial examination of the *Nodal* heterozygous cdKO embryos suggests that there may be some rescue of the HPE phenotype (Figure 7A). From 317 embryos dissected at 10.0 dpc we identified 38 *Nodal* heterozygous cdKO embryos, representing 12% of the total, which compares well to the expected 12.5% from these crosses. Other than two severely delayed embryos, and a small proportion (less than 10%) that had severe anterior truncations, the *Nodal* heterozygous cdKO embryos could be divided into two main phenotypic classes. Around one quarter of the total showed a partial rescue of the cdKO phenotype, such that the forebrain vesicle was better organized and larger in size compared to the cdKO (Figure 7A). Additionally, it appears that there is some improvement in the morphogenesis of the ventral neuroepithelium in these embryos (arrowhead, Figure 7A). The other major phenotype, seen in almost two thirds of *Nodal* heterozygous cdKO embryos was a reduction in the forebrain. *Nodal^+/z^*;cdKO embryos with a reduced forebrain also had a highly disorganized neuroepithelium (Figure 7A). These results suggest that the HPE phenotype seen in cdKO embryos can be at least partially rescued by Nodal heterozygosity, consistent with the defects being due to increased activity of the Nodal/Smad pathway.

To confirm that the Nodal heterozygous mutation was reducing expression of Smad2 target genes, we analyzed expression of *Fgf8*, which is a direct Smad2/FoxH1 target [53]. As shown earlier, *Fgf8* expression is increased in cdKO embryos (Figure 2A), whereas, *Fgf8* expression was significantly reduced in the forebrain of *Nodal^+/z^*;cdKO embryos, consistent with a reduction in Nodal signaling to Smad2 (Figure 7B). In order to determine whether reducing Nodal signaling in cdKO embryos could affect the output of the Shh signaling pathway, we analyzed the expression level of *Nkx2.1*, a target of Shh signaling in the forebrain at 9.0 dpc. Strikingly, *Nkx2.1* expression was restored in the ventral forebrain of *Nodal^+/z^*;cdKO while *Nkx2.1* expression was clearly reduced in cdKO embryos (Figure 7C). Taken together, these results suggest that Nodal signaling plays a role in regulating Shh signaling during forebrain development, and that unchecked Nodal signaling in the absence of Tgifs is responsible, at least partially, for disrupting Shh signaling in cdKO embryos.

### Tgifs coordinate Nodal and Gli3 signaling to regulate *Fgf8* expression

**Figure 8:**
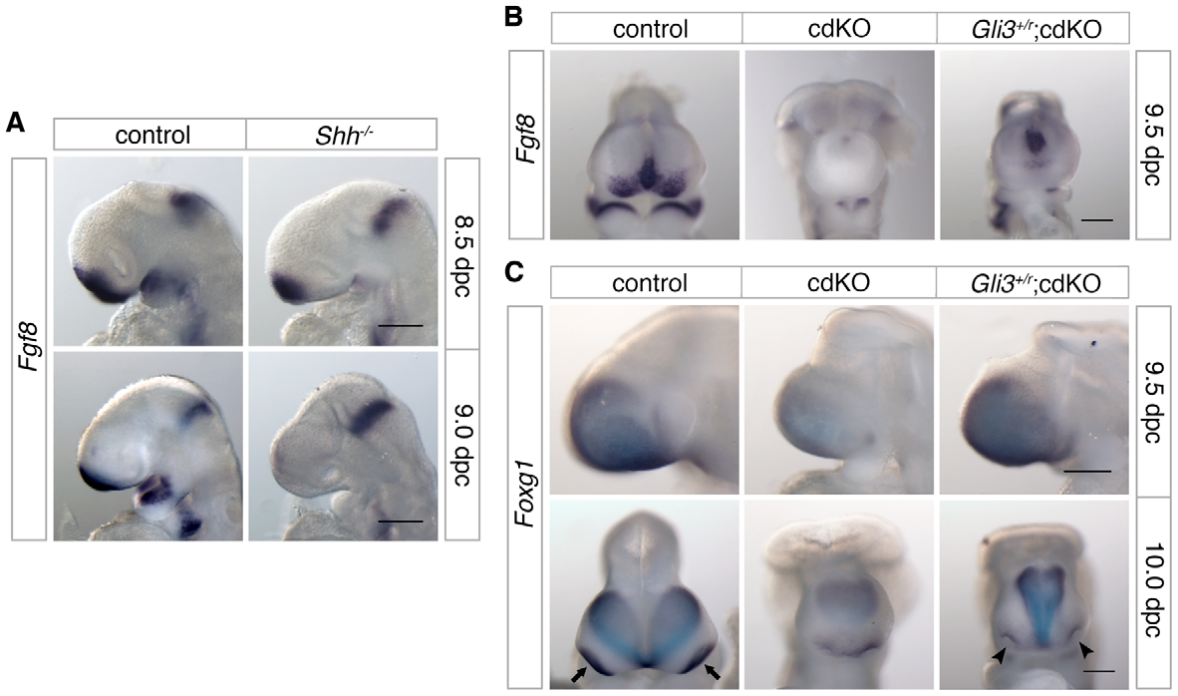
Analysis of *Fgf8* expression. (A) Control and *Shh* null embryos were analyzed for *Fgf8* expression at 8.5 and 9.0 dpc. (B) Control, cdKO and *Gli3^+/r^*;cdKO embryos were analyzed for *Fgf8* expression at 9.5 dpc, and in (C) for *Foxg1* expression at 9.5 and 10.0 dpc. Embryos are representative of at least three of each genotype at each stage and 5 each for panel B. Arrows indicate the eye field expression of *Foxg1*, and show the partial rescue of eye field separation in the *Gli3^+/r^*;cdKO embryo (arrowheads). Scale bar: 180 µm at 8.5 dpc and 250 µm at 9.0 dpc in A; 250 µm in B, C and D.

Fgf8 plays a role in coordinating multiple patterning centers during forebrain development [64], [65]. In the telencephalon, *Fgf8* is a target of TGFβ/Nodal signaling, and is also negatively regulated by Gli3, a potent inhibitory factor of Shh signaling, during early forebrain development [24]. Analysis of *Fgf8* expression in *Shh* null embryos at 8.5 dpc showed that *Fgf8* was expressed in the ventral forebrain (Figure 8A). However, consistent with previous work [64], *Fgf8* expression was reduced in the telencephalon of *Shh* null embryos at 8.5 dpc and effectively absent by 9.0 dpc (Figure 8A). In contrast to the reduction of *Fgf8* expression in the *Shh* null embryos, the cdKO forebrain at 9.0 dpc showed increased expression of *Fgf8*, most likely due to increased Nodal signaling (Figure 2A and Figure 7B). Interestingly, however, analysis at 9.5 dpc revealed that *Fgf8* expression was not maintained in cdKO embryos, while *Fgf8* expression was clearly restored in *Gli3^+/r^*;cdKO embryos (Figure 8B). This result suggests that, by 9.5 dpc, *Fgf8* expression is no longer maintained by Nodal signaling and that the excess Gli3 in the cdKO limits *Fgf8* expression. We next analyzed the expression pattern of *Foxg1*, a target of Fgf8 signaling at 9.5 dpc. *Foxg1* expression was increased in the cdKO forebrain tissue at 9.0 dpc consistent with the increased expression of *Fgf8* (see Figure 2A). At 9.5 dpc, *Foxg1* expression in the telencephalon was clearly reduced in the cdKO, whereas, the level of *Foxg1* expression was restored to levels similar to that in controls in *Gli3^+/r^*;cdKO embryos (Figure 8C). Analysis at 10.0 dpc also revealed that *Foxg1* expression was reduced in the neuroepithelium, but was partially restored in *Gli3^+/r^*;cdKO embryos. The expression of *Foxg1* in the optic vesicle was reduced and was seen as a continuous band in the cdKO (Figure 8C). Although in *Gli3^+/r^*;cdKO embryos *Foxg1* expression was lower than in controls in the optic vesicle, the expression domains were clearly better separated than in cdKO embryos, providing further evidence for a partial rescue of eye field separation (arrowheads, Figure 8C). Taken together, these results suggest that, at 9.0 dpc *Fgf8* expression is dependent on TGFβ/Nodal signaling, whereas, by 9.5 dpc the effect of TGFβ/Nodal signaling decreases and repression of *Fgf8* by Gli3 becomes more pronounced.

## Discussion

**Figure 9:**
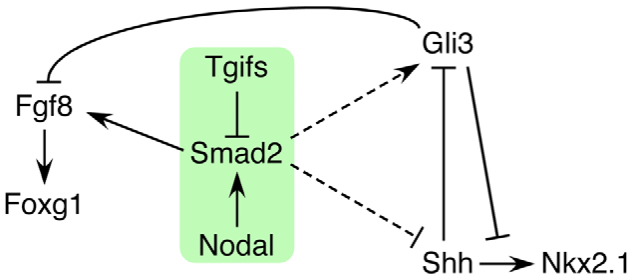
Model for the role of Tgifs in signaling during forebrain development. A tentative model is shown that describes the data presented here. Briefly, Tgifs limit Smad2 transcriptional activity, which is required for activation of *Fgf8* expression. Tgif regulation of the Nodal-Smad2 pathway is required for the correct balance between Gli3 and Shh activity in the Shh pathway. Dashed lines indicate that the links from the Nodal-Smad2 pathway to Shh signaling components may not be direct, and that the regulation may be of both *Shh* and *Gli3*, or may occur primarily via one of them.

Of the 12 genetic loci associated with HPE in humans, the best characterized (*SHH, SIX3 and ZIC2*) are all linked to the Shh pathway. In contrast, while mutations in the *TGIF1* gene, which encodes a corepressor for TGFβ/Nodal signaling, are associated with HPE pathogenesis, the underlying role of Tgif function in forebrain development has remained unclear. We now demonstrate that all embryos with a conditional epiblast-specific double knock-out of *Tgif1* and *Tgif2* exhibit early HPE-like phenotypes that are reminiscent of those seen in *Shh* null embryos. Our results provide strong evidence that a major function of Tgifs in the forebrain is to maintain the proper balance between Shh and its antagonist, Gli3, by limiting Nodal signaling. These results resolve the conundrum of how Tgif function is associated with HPE, and identify novel points of coordination between the Shh, Nodal and FGF signaling pathways during anterior development (Figure 9).

*SHH*, *SIX3*, *ZIC2* and *TGIF1*, are the four genes that are most commonly screened as a part of the genetic evaluation of human HPE patients [66]. Mice homozygous for a *Shh* null allele exhibit defects in midline facial features including cyclopia and proboscis that are typically seen in severe cases of human HPE, suggesting that *SHH* mutations do contribute to HPE in humans [50]. Recent work showed that the transcription factor Six3 is directly linked to Shh signaling by acting as a transcriptional activator of the *Shh* gene, specifically in the ventral forebrain [21], [22]. *ZIC2*, encodes a zinc-finger containing transcription factor, that has been shown to be important for forebrain patterning and Shh signaling [67], [68]. Thus, the best characterized HPE mutations appear to target the Shh signaling pathway. In contrast, the role in HPE pathogenesis of mutations in *TGIF1*, which encodes a corepressor for TGFβ/Nodal signaling, has long remained unclear. Loss of function mutations in the *Tgif1* gene in mice have no severe phenotypes in a mixed strain background, although an intragenic mutation in *Tgif1*, which may create a hypomorphic allele, has been shown to cause anterior defects in a strain specific manner [47]. However, HPE phenotypes have not been seen in *Tgif1* or *Tgif2* mutants, and these analyses have not yet shed light on any potential role in HPE pathogenesis.

Tgif2, a closely related Tgif1 paralog present in mouse and human, shares conserved functions with Tgif1 [69]. Both *Tgif1* and *Tgif2* show ubiquitous expression in the embryo proper from at least 6.0 dpc, consistent with the possibility of overlapping function during early development. As with *Tgif1* mutations, mice that carry a homozygous *Tgif2* mutation do not show appreciable phenotypes in a mixed strain background. Mice with both *Tgif1* and *Tgif2* mutations, with at least one wild-type allele of either *Tgif1* or *Tgif2*, are also viable and fertile in a mixed strain background [48]. In contrast, embryos with homozygous mutation of both *Tgif1* and *Tgif2* fail to gastrulate, providing strong evidence that Tgif1 and Tgif2 perform essential overlapping functions during embryogenesis. Thus, although there is no evidence suggesting that human *TGIF2* is associated with HPE [42], it is possible at least in mice, that both proteins share overlapping functions in anterior development. We generated embryos with *Sox2-Cre* mediated conditional deletion of *Tgif1* in the background of a *Tgif2* null, which allows the resulting embryos to undergo gastrulation successfully. At 10.0 dpc, the cdKO embryos have an HPE-like forebrain and neuroepithelium morphology, and the expression patterns of *Pax2* and *Pax7* suggest that separation of midline facial features is defective. Moreover, SEM analysis shows that separation of the ventral lips of the cephalic neural fold is defective, consistent with the failure to divide midline facial features. These phenotypes are typical of early HPE mouse mutants such as *Shh* null embryos, clearly demonstrating that Tgif1 and Tgif2 share redundant functions and together are essential players in normal forebrain development. Although the majority of cdKO embryos fail to survive past 11.0 dpc, from an analysis of 117 embryos where approximately 30 were expected to be cdKO, we were able to identify two embryos lacking both Tgif1 and Tgif2 at 12.5 dpc, which had presumably survived to this point due to a slight delay in recombination of the conditional *Tgif1* allele. Interestingly, these two embryos also showed remarkable similarity to *Shh* null embryos at the same stage. Specifically, one had a proboscis and both had cyclopia, further reinforcing the idea that the early phenotypes analyzed in detail here are clear precursors of later HPE. While the fact that relatively few embryos survive past 11.0 dpc limits our ability to analyze later HPE phenotypes in detail, those cdKO embryos that do survive to 12.5 dpc have classic HPE phenotypes. Despite the similarity of the HPE-like phenotypes, it should be noted that there are some differences between our cdKO and *Shh* null embryos. Such differences include the failure of the midbrain neural tube to close, which is not seen in *Shh* nulls, and the fact that the majority of cdKO embryos die by 11.0 dpc, whereas most *Shh* null embryos survive to late gestation. These differences aside, this work provides the first clear evidence from mouse models for a role for loss of Tgif function in HPE pathogenesis.

Our data suggest that Tgif function is required for appropriate Shh signaling during forebrain development. In cdKO embryos, *Shh* expression is present but reduced in the PrCP, and is undetectable in the neuroepithelium, suggesting that *Shh* is transcriptionally activated but that its expression is not properly maintained. In addition to the defective *Shh* expression in the forebrain, the expression of downstream targets of Shh signaling is significantly reduced in the forebrain. Expression of *Gli3*, which encodes a repressor for the Shh signaling pathway in the forebrain, is up-regulated in *Shh* null embryos, and the HPE phenotype of *Shh* null embryos is partially rescued when the genetic dose of *Gli3* is reduced [27], [61]. Similarly, cdKO embryos showed an increased level of *Gli3* expression in the forebrain. Intriguingly, the increase in *Gli3* expression in cdKO was clearly higher than in *Shh* null embryos, suggesting that there is an additional, Shh-independent, Tgif-dependent mechanism that regulates *Gli3* gene expression. In cdKO embryos with a reduced dose of *Gli3*, there was a phenotypic rescue in the morphology of the forebrain neuroepithelium and also of the craniofacial features. Additionally, *Nkx2.1* expression was restored in the ventral diencephalon of cdKO embryos carrying a *Gli3* heterozygous mutation, while, in agreement with previous work, there was no rescue of *Nkx2.1* expression in the diencephalon of *Shh* null embryos with a *Gli3* heterozygous mutation [61]. This suggests that some level of *Shh* expression is required for *Nkx2.1* expression, and also suggests that sufficient *Shh* expression is present to activate *Nkx2.1* in the ventral diencephalon of cdKO embryos. However, it should be noted that *Shh* expression was not rescued in the ventral forebrain of *Gli3* mutant cdKO embryos. Although many mutations that cause HPE may do so by affecting the Shh pathway, and specifically the balance between Shh and Gli3, it is worth pointing out that *Gli3* heterozygosity does not rescue all mouse models of HPE. For example, the phenotype of *Fgfr1;Fgfr2* double mutant embryos is not rescued by *Gli3* mutation, suggesting that there is some specificity to the rescue by *Gli3* mutations [29]. Taken together, these data provide strong evidence that *Tgifs* play a critical role in regulating Shh signaling during forebrain development, and that the loss of Tgif-mediated regulation of the Shh pathway is important for HPE pathogenesis.

Studies in humans and mice have implicated both the retinoic acid and TGFβ/Nodal pathways in HPE pathogenesis. Retinoic acid mediated teratogenesis in humans is known to contribute to CNS anomalies such as hydrocephalus, and in a few rare cases, HPE, and in mice *in utero* administration of retinoic acid to pregnant females on gestational day 7 leads to embryos with severe craniofacial phenotypes including HPE [70], [71]. However, mutations in genes associated with retinoic acid signaling have not been identified in HPE patients. Mutations that likely reduce the output of the TGFβ/Nodal pathway have been found in human patients with HPE or laterality defects. Mutations in *TDGF1* (also referred to as *CRIPTO*), an EGF-CFC family member that acts as a co-factor for the NODAL ligand, and in the gene encoding the forkhead transcription factor FOXH1 (also known as FAST1), which complexes with SMAD2 and SMAD4 to mediate TGFβ/NODAL signaling, have been identified [72], [73]. However, these mutations are found very rarely in HPE, and in general are not complete loss of function alleles. Studies in *Tdgf1* null and *Foxh1* null embryos show that these genes are required for the activity of the early organizing centers during gastrulation [74], [75]. In *Tdgf1* null embryos, marker analysis shows that expression of organizer genes including *Brachyury*, *Cerl1* and *Lhx1* is defective. Similarly in *Foxh1* null embryos, expression of organizer genes such as *Foxa2* and *Goosecoid*, is reduced, and analysis of forebrain markers such as *Six3*, *Hesx1* and *Fgf8* shows that the forebrain tissue is significantly reduced, exhibiting a mild anterior truncation phenotype [75]. It has also been suggested that *Nodal;Smad2* double heterozygous mutations can result in HPE, again indicating that a reduction in TGFβ/Nodal signaling is important in HPE pathogenesis [1]. However, the morphology of these embryos suggests that in most cases forebrain tissue is reduced or missing, rather than exhibiting a clear HPE phenotype as seen in *Shh* null embryos, for example. Thus it appears that, at least in mice, a reduction in the TGFβ/Nodal signaling pathway primarily results in defective early organizing centers, leading to phenotypes such as a small or truncated forebrain. In contrast, in our cdKO embryos, marker analysis shows that the organizing centers are formed, and that the forebrain does not show an anterior truncation phenotype. In addition, the forebrain morphology shows an HPE phenotype that is similar in many respects to that seen in *Shh* null embryos, and forebrain markers show relatively normal expression patterns, suggesting that the forebrain is reasonably formed in cdKO embryos. Our own analysis of embryos that are heterozygous for both *Smad2* and *Nodal* is in agreement with the idea that HPE is relatively rare in this genetic combination – only one out of 41 double heterozygotes analyzed at 10.5–12.5 dpc had HPE, with an additional 15 showing severe growth delays or anterior truncations. Additionally, it is interesting to note that the comparison of cdKO, *Shh* null and *Smad2/Nodal* double heterozygous embryos with HPE at 12.5 dpc suggests that, at least superficially, the *Shh* null and cdKO are more similar to each other than to the *Smad2/Nodal* double heterozygote. Thus the loss of *Tgif1* and *Tgif2* causes a classic HPE phenotype, rather than the predominance of anterior truncations that are seen in embryos with reduced activity of the TGFβ/Nodal pathway.

Our results, together with evidence from mouse mutants with reduced Nodal activity, support a model in which decreased Nodal signaling primarily results in a truncation of anterior tissues, whereas increased Nodal signaling (as in our cdKO embryos) causes classic HPE phenotypes. One alternate interpretation of this difference between the HPE phenotype in cdKO embryos and other TGFβ/Nodal mouse mutants is that the effects of loss of Tgif function are independent of TGFβ/Nodal signaling during forebrain development. However, we have shown that embryos that are homozygous null for both *Tgif1* and *Tgif2* fail gastrulation, and that the gastrulation defect is dependent on increased TGFβ/Nodal signaling. Similarly, left-right asymmetry defects in cdKO embryos can be partially rescued by reducing the dose of *Nodal* [48]. Here we show that at 9.0 dpc, *Fgf8* expression is increased in the cdKO, consistent with the derepression of a Smad/Foxh1 target gene [53]. Importantly, this excess *Fgf8* expression is reduced in the *Nodal* heterozygote. Reducing the dose of Nodal also results in a partial rescue of the HPE phenotypes in a proportion of cdKO embryos. Most of the remaining *Nodal* heterozygous cdKO embryos have a mild anterior truncation, which might indicate that there are additional Nodal and Tgif specific phenotypes, but could also reflect the effect of mutating multiple components of the Nodal pathway. However, with the restoration of *Nkx2.1* expression in the *Nodal* heterozygous cdKO forebrain, this is clearly consistent with a model in which Tgifs limit Nodal signaling and that the absence of this restraint causes disruption of the Shh pathway and HPE. It should, however, be noted that we have not yet exhaustively analyzed the Shh signaling pathway in *Nodal* heterozygous cdKO embryos, and it will clearly be of interest in the future to determine precisely how *Nodal* heterozygosity rescues *Nkx2.1* expression and forebrain morphology. One attractive candidate for the Nodal target would be the *Gli3* gene, given its striking upregulation in the cdKO. However, this remains to be tested and potential effects of other pathways, such as FGF signaling, that specify forebrain patterning should also be considered. On balance, it is reasonable at this point to suggest that the HPE phenotype seen in cdKO embryos is dependent on excessive TGFβ/Nodal signaling due to the loss of Tgif-mediated repression, and that disruption of the Shh pathway makes a major contribution to the phenotype.

The increased *Fgf8* expression seen at 9.0 dpc in cdKO embryos is consistent with an increase in Nodal signaling, and is in fact reduced in the *Nodal* heterozygote. However, this also appears to be somewhat at odds with the increased *Gli3* expression seen in cdKO embryos, since Gli3 represses *Fgf8* expression in the anterior. However, by 9.5 dpc, we show that *Fgf8* expression in the cdKO telencephalon is essentially lost, consistent with increased repression by Gli3. It is likely that by this stage the effect of Nodal signaling is diminishing, even in the cdKO, and so the excess Gli3 predominates. In support of this, *Gli3* heterozygosity restores some *Fgf8* expression and restores expression of *Foxg1*, which is a downstream target of FGF signals in the anterior [65]. Analysis of *Fgf8* expression in *Shh* null embryos reveals that expression is already lost by 9.0 dpc, while at this stage in the cdKO it is increased. However, as with the *Gli3* heterozygous cdKO at 9.5 dpc, the loss of *Fgf8* expression in *Shh* null embryos can be rescued by *Gli3* heterozygosity [24], [61]. Thus the loss of *Fgf8* expression in the anterior may contribute to the HPE phenotypes seen in both *Shh* null and cdKO embryos, and the difference in timing of the loss of expression may also be in part responsible for some of the differences between these two models. Given that loss of *Fgf8* expression is common to the *Shh* null and cdKO HPE models, it is tempting to speculate that in the small proportion of *Smad2/Nodal* double heterozygous mutants with the HPE phenotype is in part due to a failure to fully activate *Fgf8* expression.

Taken together, our data suggest a model in which Tgifs limit the activity of the Nodal-Smad2 pathway, which is required for full activation of Smad/Foxh1 targets, such as *Fgf8* (Figure 9). In addition we provide evidence that regulation of Nodal signaling by Tgifs is required to maintain the appropriate balance between Shh and Gli3 levels in the forebrain. However, it should be noted that we do not yet know whether this occurs via direct regulation of *Gli3* or *Shh* expression (dashed lines in Figure 9), or whether the regulation is less direct. An additional possibility is that at least some of the regulation of the Shh pathway by Tgifs is independent of Nodal/Smad2. For example, *Gli3* might be a direct target of Tgif repression, although the rescue of *Nkx2.1* expression in the *Nodal* heterozygotes is consistent with a Nodal dependent regulation of the Shh pathway. In summary, this work provides the first clear evidence for a role for loss of Tgif function in HPE pathogenesis, and suggests that Tgifs regulate Shh signaling pathway activity. We propose that Tgif function limits *Gli3* expression, and that by a mechanism that is independent of changes in Gli3 levels, Tgifs are required for full *Shh* expression in the PrCP and neuroepithelium. Thus, the Tgifs have significant contributions to HPE pathogenesis by functioning as key regulators of Shh signaling during forebrain development, most likely by limiting Nodal signaling.

## Materials and Methods

### Ethics statement

All animal procedures were approved by the Animal Care and Use Committee of the University of Virginia, which is fully accredited by the AAALAC.

### Mice and DNA analysis

The *loxP* flanked *Tgif* allele [45], *Tgif2 null* [48], *loxP* flanked *Gli3* allele [62], *Nodal* mutants [76], *loxP* flanked *Smad2* allele [77], and the *Sox2-Cre* line [49] have been described previously. Conditional *Shh* mice were obtained from Jackson labs (stock 4293; [78]). The *Gli3*, *Shh* and *Smad2* alleles each contain loxP flanked exons, which when recombined result in null alleles, and are referred to here as ‘r’ for recombined (null). The *Nodal* null allele is referred to as ‘z’, for an introduced lacZ reporter. All mouse lines were maintained on a mixed C57BL/6J×129Sv/J background. Genomic DNA for PCR genotype analysis was purified from ear punch, at post-natal day 21 (P21), or yolk sac (7.0–10.0 dpc) by HotShot [79].

### *In situ* hybridization

Whole-mount in situ hybridization was performed on 7.5–10.0 dpc embryos with digoxigenin-labeled riboprobes, as described [80]. Stained embryos were processed for sectioning and histology as described [58]. All images are representative of at least three embryos analyzed.

### Histology, immunohistochemistry (IHC), and whole-mount analysis

Embryos were fixed overnight in 4% paraformaldehyde at 4°C, dehydrated through an ethanol series (70%, 90%, 95%, 100% ×2 for 30 minutes each), incubated in xylene twice for 60 minutes and 1∶1 xylene/paraffin for 60 minutes at 60°C, then embedded in paraffin wax, and sectioned at 7 µm. For Hematoxylin and Eosin (H&E) histological analysis, sections were de-paraffinized with xylene and stained with H&E. Multiple sections per embryo were incubated with primary antibodies for pHH3 or active caspase 3 as described [48]. For IHC, antibody staining was detected using Vectastain ABC (Vector Laboratories) and developed with Impact DAB (Vector Laboratories). For H&E and IHC images were captured using an Olympus BX51 microscope and either an Olympus SZX12 or DP70 digital camera, and manipulated in Adobe Photoshop. Images of 7.0–10.0 dpc embryos were captured using a Leica MZ16 stereomicroscope and QImaging 5.0 RTV digital camera.

### Scanning electron microscopy

Embryos were fixed overnight in 4% paraformaldehyde at 4°C, and then fixed with osmium tetraoxide for 30 min and dehydrated through an ethanol series (40%, 60%, 80% and 100% ×2 for 15 minutes each). Dehydrated samples were further processed in an Autosamdri-815 (Tousimis Research Corporation) and were gold coated by using a SCD005 Sputter Coater (Bal-Tec). Images were captured using a JSM-6400 Scanning Electron Microscope (JEOL).

## Supporting Information

Figure S1 Comparison of a *Nodal;Smad2* double heterozygous embryo with HPE to Shh null and cdKO embryos. Whole mount images and H&E stained sections of fixed and paraffin-embedded control, cdKO and *Shh* null embryos at 12.5 dpc are shown (note these are the same images as in Figure 1E). Additionally, similar images of a *Nodal;Smad2* double heterozygote are shown to the right. The two planes of section are indicated in the upper panels, and a magnified view of the eye is shown at the bottom. Note that the eyes in the *Nodal;Smad2* double heterozygote have formed and begun to separate, whereas the *Shh* null and cdKO have a single eye rudiment. The *Nodal;Smad2* double heterozygote was the only embryo with HPE from 41 of this genotype examined at 10.5–12.5 dpc. Scale bar: 2 mm for whole-mount; 250 µm for *i*, 500 µm for *ii* and 100 µm for *ii*-zoom. In the lower panels, the eye in the control embryo is bracketed, the single eye fields in the cdKO and *Shh* null are circled, and the partial separation between the two eyes in the *Nodal;Smad2* double heterozygote is indicated with an arrowhead.
(TIF)
