# Supplementary figures and images for: Loss of Tgif Function Causes Holoprosencephaly by Disrupting the Shh Signaling Pathway
Source: PLoS Genet. 2012 Feb 23;8(2):e1002524. doi: 10.1371/journal.pgen.1002524 (PMC3285584; doi:10.1371/journal.pgen.1002524)

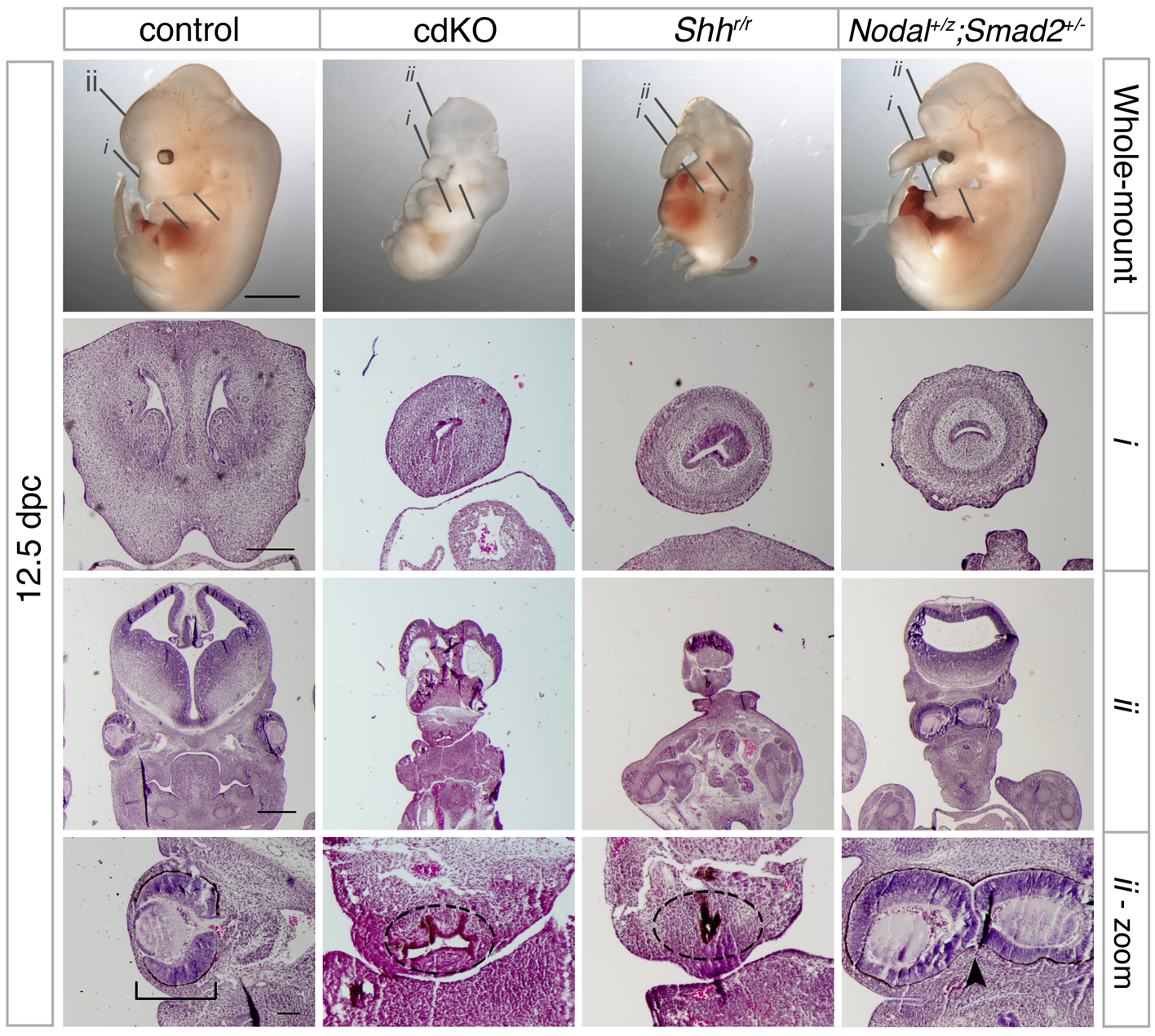

Supplement: Figure S1 — Comparison of a Nodal;Smad2 double heterozygous embryo with HPE to Shh null and cdKO embryos. Whole mount images and H&E stained sections of fixed and paraffin-embedded control, cdKO and Shh null embryos at 12.5 dpc are shown (note these are the same images as in Figure 1E). Additionally, similar images of a Nodal;Smad2 double heterozygote are shown to the right. The two planes of section are indicated in the upper panels, and a magnified view of the eye is shown at the bottom. Note that the eyes in the Nodal;Smad2 double heterozygote have formed and begun to separate, whereas the Shh null and cdKO have a single eye rudiment. The Nodal;Smad2 double heterozygote was the only embryo with HPE from 41 of this genotype examined at 10.5–12.5 dpc. Scale bar: 2 mm for whole-mount; 250 µm for i, 500 µm for ii and 100 µm for ii-zoom. In the lower panels, the eye in the control embryo is bracketed, the single eye fields in the cdKO and Shh null are circled, and the partial separation between the two eyes in the Nodal;Smad2 double heterozygote is indicated with an arrowhead. (TIF) [file pgen.1002524.s001.tif]
